# Transfer, integration, and inverse design of metasurfaces in suspended membranes

**DOI:** 10.1007/s44275-026-00041-y

**Published:** 2026-02-16

**Authors:** Nanzhong Deng, Yue Xiao, Srilok Srinivasan, Subramanian K. R. S. Sankaranarayanan, Xu Zhang, David Czaplewski, Daniel Lopez, Haogang Cai

**Affiliations:** 1 Tech4Health Institute, New York University Grossman School of Medicine, Queens, NY 11101 USA; 2 Department of Biomedical Engineering, New York University, Brooklyn, NY 11201 USA; 3 Center for Nanoscale Materials, Argonne National Laboratory, Lemont, IL 60439 USA; 4 Department of Mechanical and Industrial Engineering, University of Illinois, Chicago, IL 60607 USA; 5 Department of Electrical and Computer Engineering, Carnegie Mellon University, Pittsburgh, PA 15213 USA; 6 Department of Electrical Engineering & Materials Research Institute, The Pennsylvania State University, University Park, PA 16802 USA; 7 Department of Radiology, New York University Grossman School of Medicine, New York, NY 10016 USA

**Keywords:** Metasurface, Optical fiber, Transfer, Micro-punching, Inverse design

## Abstract

Despite the vast promise of abrupt wavefront engineering within subwavelength thickness, most optical metasurfaces are still bound to bulky and rigid substrates. Recently, metasurfaces in suspended membranes (MISMs) have attracted increasing attention due to their unique flexible, conformal properties and their ability to minimize undesired substrate effects. Most importantly, the MISM platform enables metasurface transfer and integration with non-conventional substrates and electronic/photonic devices. By summarizing multiple approaches to create MISMs with a variety of membrane and sacrificial layer materials and configurations, we demonstrate the Omni-Purpose Transfer and Integration of Metasurfaces in Suspended Membranes (OPTIMISM), overcoming the existing limitations on metasurface geometries or materials. It is particularly suitable for metasurface integration on optical fiber tips to form meta-optic probes for broad applications, including biomedical and endoscopic imaging and sensing. Considering the various configurations of membrane dielectric environment in integrated MISM devices, we performed a systematic investigation to demonstrate the strong influence of the surrounding refractive index on ultrathin metasurface design based on both conventional forward design (library search) and inverse design strategy (evolutionary algorithm). Our findings highlight the advantage of the inverse design strategy leveraging meta-atom non-local interactions, and the great potential of the MISM platform for universal and scalable metasurface transfer and integration.

**Supplementary Information:**

The online version contains supplementary material available at 10.1007/s44275-026-00041-y.

## Introduction

Despite the vast promise of abrupt wavefront engineering within subwavelength thickness, most optical metasurfaces are still bound to bulky and rigid substrates, which limits the full exploration of their potential across the broad optical spectral range from deep ultraviolet [1], visible [2], to terahertz [3]. Decoupled from conventional bulky and rigid substrates, metasurfaces in suspended membranes (MISMs) have drawn increasing attention due to their unique advantages. First of all, optically “free-floating” dielectric metasurfaces on membranes with a relatively low refractive index (e.g., Al_2_O_3_) [4] provide both higher operational efficiencies and higher resonance Q factors by minimizing undesired substrate effects. In fact, it has roots in a long history of plasmonic metasurfaces. For example, etching cavities through a gold nanohole array was found to significantly improve the extraordinary optical transmission (EOT) signal and sensitivity [5]. “Free-floating” gold nanohole arrays on SiN_*x*_ membranes were demonstrated for biosensing applications before the emergence of dielectric metasurfaces [6, 7]. Secondly, “flexible” metasurfaces embedded in elastomer membranes can be used for dynamic actuation. For example, polydimethylsiloxane (PDMS)-embedded metasurfaces were mechanically stretched for tunable focusing [8], holograms [9], and structural color [10]. Metasurfaces were also integrated with dielectric elastomer actuators, composed of transparent polyacrylate elastomers and stretchable carbon nanotube electrodes, for electrical actuation [11]. Finally, MISMs facilitate the transfer-enabled integration of metasurfaces with non-conventional substrates or electronic/photonic devices, which is not possible using current fabrication processes. For example, flexible elastomer MISMs were integrated conformally on nonplanar surfaces to decouple the optical function from topography [12]. Thin-film MISMs were attached to goggle and eyewear surfaces, e.g., meta-holograms for visual reporting [13–15]. In this work, we will focus on the integration of metasurfaces on optical fiber tips to form meta-optic probes, which improve the compactness and precision for broad applications including biomedical and endoscopic imaging [16–21] and sensing [22, 23].

To achieve a subwavelength feature size, optical metasurfaces mostly rely on electron beam lithography (EBL). The multi-step fabrication processes (resist spin-coating, EBL, material deposition, lift-off, and etching) are usually performed on bulky substrates (e.g., Si, glass wafers) and are not compatible with non-conventional substrates (e.g., optical fiber tips). In previous studies, metasurfaces patterned separately on glass substrates were either manually assembled with optical fibers [17, 18] or glued onto the fiber tips [16]. Direct patterning on fiber tips has been demonstrated by alternative lithography techniques, including two-photon polymerization (TPP) [19, 20], focused ion beam (FIB) [21, 22], nanosphere lithography [23], and nanoimprint lithography (NIL) [24], which have respective constraints on metasurface geometries or materials (e.g., nanoholes in plasmonic metals by FIB, polymers by TPP). There is a pressing need for a novel technology that transfers metasurfaces with more design freedom (based on EBL) from conventional donor substrates to non-conventional receiver substrates. However, existing MISM transfer processes are limited to nanohole membrane metasurfaces [25, 26] or plasmonic metasurfaces [8–10, 13, 14, 27], while dielectric metasurfaces (Si nanopillars) are limited to thick elastomer membranes for dynamic actuation [11, 12] (see Table 1). Our previous work demonstrated the transfer of dielectric meta-atoms (TiO_2_) embedded in thin membranes (poly(methyl methacrylate) (PMMA)) for the first time [28].

**Table 1:** Comparison of MISM membrane, sacrificial layer, etchant, metasurface, transfer, and integration

Reference		Membrane	Sacrificial layer	Etching	Metasurface	Transfer process	Integrated device
Nanohole membrane	[25]	Si	SiO_2_	HF	Si nanoholes (i.e., membrane layer)	Elastomer stamp (PDMS)	Ultraspectral imaging chip (metasurfaces as spectral filters)
	[26]	PMMA	SiO_2_	HF	PMMA nanoholes (i.e., membrane layer)	Micro-punching	Metalens on fiber tip
Plasmonic	[27]	PMMA, OrmoClear	Cr	Cr etchant	Au Fresnel zone plate	Adhesive tape, optical adhesive (NOA 68)	Metalens on fiber tip
	[13, 14]	SU-8	OmniCoat	Microposit MF-319	Au nanorods	SU-8 (i.e., membrane layer)	Meta-holograms on nonplanar surfaces
Plasmonic+elastomer	[8–10]	PDMS	HSQ on PMMA	None	Au nanorods, Al nanoparticles	PDMS (i.e., membrane layer)	Metasurfaces with mechanical actuation
Elastomer membrane	[12]	PDMS	Ge	NH_4_OH:H_2_O_2_:H_2_O (1:1:30)	Si nanopillars	PDMS (i.e., membrane layer)	Metasurfaces on nonplanar surfaces
	[11]	Polyacrylate elastomer	GeO_2_	H_2_O	Si nanopillars	Polyacrylate elastomer (VHB Tape 4905, i.e., membrane layer)	Metalens with electrical actuation
OPTIMISM proposed in this work		Al_2_O_3_	Si	Si etchant or dry etching	No limitation on metasurface geometry or material	Universal transfer processes (printing/adhesive or punching)	Omni-purpose applications, e.g., metasurfaces on electronic/photonic devices, fiber tips
		SU-8	OmniCoat	Microposit MF-319			
		PMMA [28]	GeO_2_	H_2_O			

**Fig. 1:**
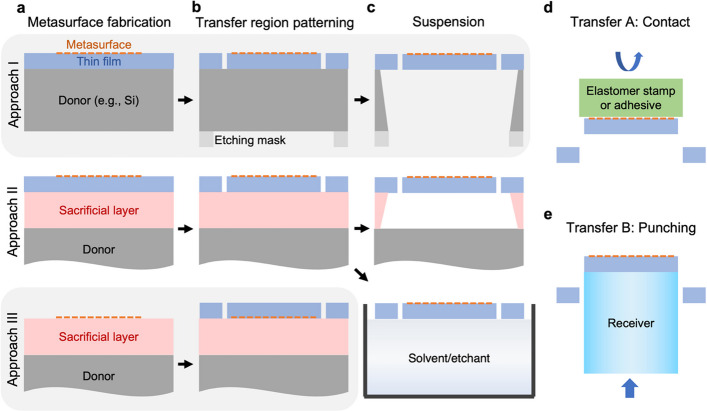
Schematic summary of the universal OPTIMSIM process. **a** Metasurface fabrication on donor substrates. **b** Transfer region patterning through additional lithographic steps. **c** Suspension-tether structure formation by etching substrates or sacrificial layers. **d** Transfer based on the printing process or using adhesives. **e** Transfer based on the micro-punching process

Here, we further propose comprehensive and universal strategies termed Omni-Purpose Transfer and Integration of Metasurfaces in Suspended Membranes (OPTIMISM, see Fig. 1), which overcome the existing limitations on metasurface geometries or materials. Metasurfaces are created following the conventional nanofabrication processes on donor substrates (see Fig. 1a); the MISM transfer regions (suspension and tether structures) are defined by an additional lithographic step with alignment (see Fig. 1b); MISMs are suspended by etching the substrates or sacrificial layers (see Fig. 1c) and then transferred through either printing (see Fig. 1d) or punching (see Fig. 1e) processes. Without using any intermediate carriers such as elastomer stamps or adhesives, the micro-punching process is simple and particularly suitable for optical fiber receivers, but it has only been used for all-polymer metasurfaces of nanohole arrays [26]. In our OPTIMISM process, there is no limitation on metasurface geometry (nanoholes, meta-atoms) or materials (plasmonic, dielectric, polymer), while the membranes can be low-index dielectric materials or polymer/resist membranes (e.g., Al_2_O_3_, PMMA, SU-8, hydrogen silsesquioxane (HSQ)).

Moreover, the polymer/resist transfer membranes can be either removed by oxygen plasma (PMMA), leaving the metasurfaces intact on receiver substrates, as reported in our previous work [28], or kept as protection coatings (SU-8, HSQ) or spacers (PMMA, OrmoClear) for multi-layer metasurface stacking [26, 27]. Considering the various configurations of the membrane dielectric environment in integrated MISM devices, we performed a systematic investigation into the influence of the surrounding refractive index on the transmission and phase outputs of meta-atom libraries for metasurface design. Taking TiO_2_ metasurfaces with HSQ coatings as an example, both high-aspect-ratio waveguide-type and ultrathin (thickness *t* ~ 1/5 of the wavelength *λ*) resonator-type metasurfaces were studied by computational simulations (COMSOL, Lumerical). It was found that waveguide-type meta-atoms are more robust, while ultrathin resonators are more sensitive to the surrounding refractive index, which was also experimentally validated. In our previous work, we demonstrated inverse design strategies to improve the optical efficiency of ultrathin resonant metasurfaces with non-local interactions [29]. We further investigate the role of the surrounding refractive index in improving metasurface performance by inverse design versus forward design.

## Results

### Metasurface transfer and integration

As a versatile platform technology, MISMs can be fabricated and transferred through various approaches (see Fig. 1). The two modules of MISM fabrication and transfer can be combined in an arbitrary manner by selecting available options based on the desired metasurface and receiver configurations. In the fabrication module approach I, metasurfaces are fabricated on thin films of a relatively low refractive index (e.g., Al_2_O_3_) deposited on donor substrates (e.g., Si wafers). The Si wafers are back-etched to suspend the Al_2_O_3_ membrane [4]. In approaches II and III, instead of substrate etching, sacrificial layers and the corresponding etchants are used. The metasurfaces are either fabricated on thin films deposited on the sacrificial layer (approach II) or directly fabricated on sacrificial layers and then embedded in thin films (approach III) [28]. MISMs are either suspended on-chip by wet or vapor etching, or fully released at the liquid–air interface and then picked up and suspended by a frame holder.

In the transfer module, approach A is contact transfer (see Fig. 1d). Similar to the contact printing processes developed for the heterogeneous hybrid integration of photonic devices [30, 31], an intermediate carrier such as an elastomer stamp is used to attach the suspended MISM from the top, breaking the tethers during a rapid retraction. Subsequently, the MISM attached to the stamp is transferred to the receiver substrate after a slow retraction. The attachment of MISM to a stamp and its release are carefully controlled by the retraction speed, taking advantage of the flexible deformation of elastomer stamps. Alternatively, heat treatment or adhesives can be used to adjust the adhesion strength between MISMs and intermediate carriers versus receiver substrates [32]. For plasmonic metasurfaces, the suspended structures can be defined simply by lithography and development processes without any etching process [8–10]. The transfer can be done even without any suspended structures, simply taking advantage of the poor adhesion between Au and Si substrates [32]. The integration of plasmonic nanopatterns on optical fiber tips was demonstrated with elastomer stamps [33] or adhesives (epoxy [34], optical adhesives [27]), which can be extended to dielectric metasurfaces using our OPTIMISM process.

**Fig. 2:**
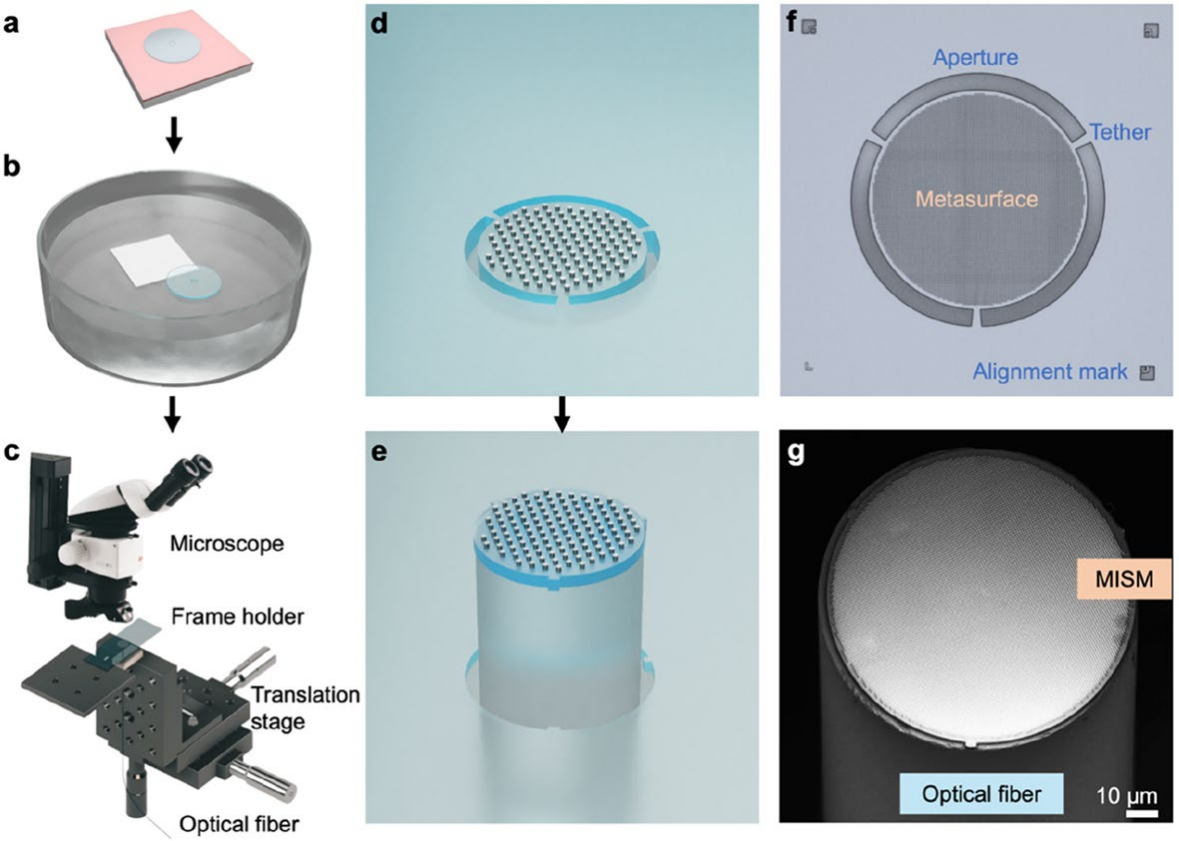
OPTIMISM punching process integrating metasurfaces on optical fiber tips. Schematic diagrams of **a** MISM fabrication on a sacrificial layer, **b** MISM release in the sacrificial etchant solution, floating at the water–air interface, and **c** micro-punching process with fiber alignment on a translation stage under a microscope. Zoom-in schematics of **d** free-standing MISM and **e** MISM on the fiber tip after punching. **f** Optical microscope image of the free-standing MISM. **g** SEM image of the MISM integrated on the fiber tip

In the transfer module, approach B comprises direct punching without using any intermediate carriers or adhesives (see Figs. 1e and 2), which is particularly suitable for optical fibers. As a proof of concept, a metasurface was fabricated on a sacrificial layer (see Fig. 2a). Aperture and tether structures were patterned in the membrane by an additional lithography step, with the metasurface aligned to the center of the aperture. The MISM was fully released in the sacrificial etchant solution and then transferred to deionized (DI) water (see Fig. 2b). MISM floating at the water–air interface can be directly picked up by receiver substrates, similar to the water transfer printing process. The patterns can be aligned in a limited time window before the residual water between the MISM and receiver substrate is dried. Here, in order to improve the alignment precision and scalability, the floating MISM was first picked up by a frame holder (e.g., a glass slide with a drilled hole, see Fig. 2c) and suspended over an opening for the punching process. Under the microscope, an optical fiber attached to a translation stage was aligned to the aperture (see Fig. 2d). During the punching process, the fiber passed through the aperture, breaking the tethers. The MISM was detached from the membrane and attached to the top of the fiber tip (see Fig. 2e). The MISM aperture and tether structures with lithographic alignment marks (for the alignment between the metasurface and aperture) are shown in the optical microscope image (see Fig. 2f). An integrated metasurface on the fiber tip was successfully demonstrated as shown in the scanning electron microscopy (SEM) image (see Fig. 2g). COMSOL simulation of the pressure distribution on an SU-8 membrane shows that the pressure is mainly concentrated on the thin tether connection parts touching the fiber edge, when an optical fiber is punched through (see Fig. S1). The tethers easily break during the punching process without affecting the metasurfaces on the membrane. The adhesion between the MISM and fiber tip was further improved by heat treatment.

**Fig. 3:**
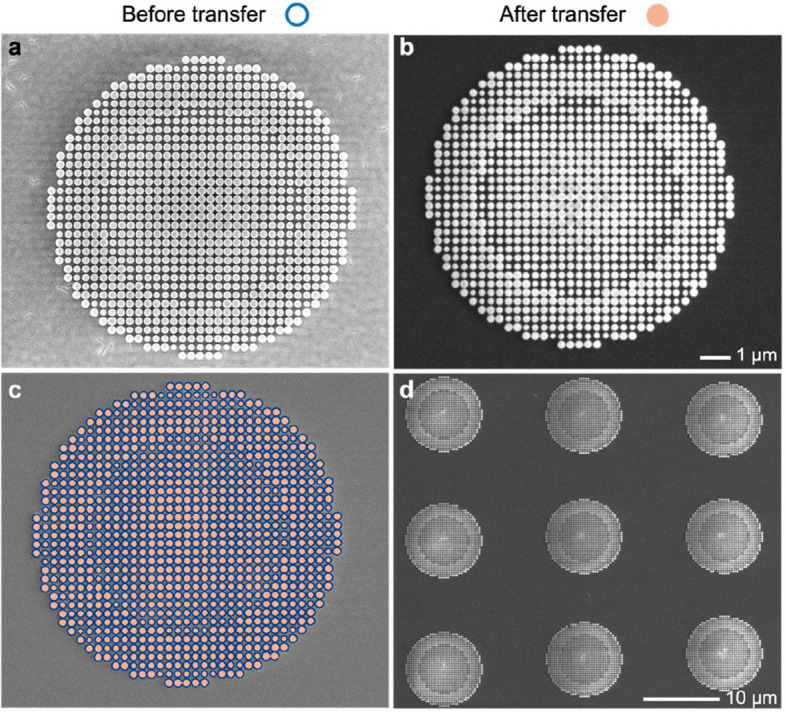
Metasurfaces before and after transfer. **a** SEM images of a metalens before transfer on the GeO_2_ sacrificial layer and **b** after transfer onto a SiN_*x*_ membrane. **c** Comparison of the meta-atom positions before and after transfer. **d** Transferred metalens array on a single membrane

To observe the transferred metasurfaces more closely, we analyzed the SEM images of a TiO_2_ metalens on the GeO_2_ sacrificial layer (see Fig. 3a) and after transfer onto a SiN_*x*_ membrane (see Fig. 3b). The overlay image shows that the position of individual meta-atoms matches very well before and after transfer (see Fig. 3c). Maintaining high pattern fidelity, the OPTIMISM process is robust, repeatable, and scalable. The transfer of metalens arrays onto a membrane was demonstrated (see Fig. 3d). Combined with an array of optical fibers, the punching process (see Fig. 2) can be extended from a single fiber to multiple fibers in parallel, pointing to potential high-throughput manufacturing. The metasurface arrays can be patterned according to the predetermined positions of optical fiber arrays, so that the global alignment of arrays will ensure the accurate alignment of individual meta-fibers.

### Meta-atom library affected by the dielectric environment

**Fig. 4:**
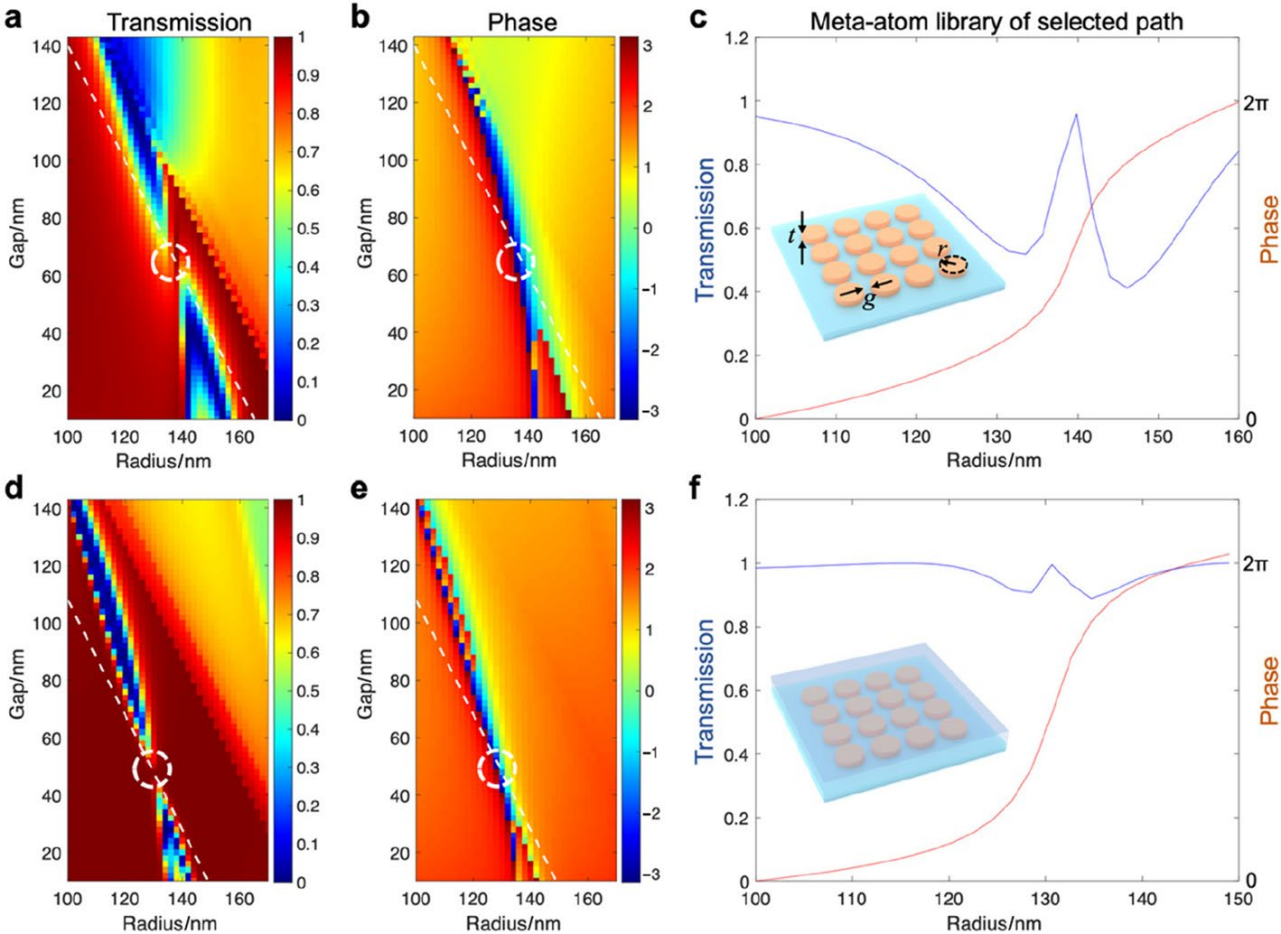
Effects of dielectric environment on ultrathin resonant metasurfaces. Simulation results of the transmission and phase outputs of a uniform wavefront of light after propagation through a homogeneous and periodic square array (circular TiO_2_ nanodiscs, thickness *t* = 120 nm, on a glass substrate). Metasurface exposed to air: **a** transmission and **b** phase as a function of the disc radius and edge-to-edge gap; **c** transmission and phase for a meta-atom library with a constant pitch of 340 nm and varying disc radius (following the path marked by white dashed lines in **a** and **b**). Metasurface embedded in the HSQ layer: **d** transmission and **e** phase as a function of the disc radius and edge-to-edge gap; **f** transmission and phase for the same library with constant pitch (308 nm) and varying disc radius

As mentioned previously, the membrane material has a broad selection from low-index dielectric materials to polymers and resists, which can be either removed or kept after the transfer process. Therefore, metasurfaces are either exposed to air on the receiver substrates or embedded in membrane spacers and coatings, which are distinct dielectric environments with significant influence on the meta-atom resonances and the whole library for metasurface design. In fact, we created dynamic tunable metasurfaces and meta-holograms, taking advantage of the multi-state outputs modulated by the surrounding medium [35]. Our previous work demonstrated that high-aspect-ratio nanopillar meta-atoms are relatively independent of each other as the fields are confined by the waveguide effect, while ultrathin resonator nanodiscs are strongly coupled with non-local interactions. Here, we further investigate the influence of the dielectric environment (e.g., air vs. HSQ) on the two different types of metasurfaces, using computational simulations (COMSOL, Lumerical). We built a TiO_2_ meta-atom library on a glass substrate by calculating the transmission and phase outputs with varying geometric parameters (radius *r*, edge-to-edge gap *g*, and thickness *t*) in periodic boundary conditions, under illumination along its axis of symmetry (*z*-axis). Based on the same circular geometry, high-aspect-ratio waveguide-type meta-atoms are nanopillars with a constant thickness of 600 nm, while ultrathin resonators are nanodiscs with a constant thickness of 120 nm (see Fig. 4).

For ultrathin resonant metasurfaces, the transmission and phase data are dependent on the in-plane geometry, which are different between air (see Fig. 4a–c) and the HSQ environment (see Fig. 4d–f). When exposed to air, the optimal meta-library selection follows a path (pitch *p* = 2*r* + *g* = 340 nm) that passes through the Huygens condition (white dashed lines and circles in Fig. 4a, b): the electric dipole and magnetic dipole spectral positions are matched, canceling back-scattering light by both dipoles and leaving only forward propagation. The selected meta-atom library provides both high transmission and a full 2π phase modulation (see Fig. 4c). As we expected, the resonances and optical outputs are strongly influenced by the surrounding refractive index. When the surrounding refractive index changes from air to HSQ, the low-transmission resonance bands become narrower and shift toward smaller (*r*, *g*) pairs in the geometric parameter space (see Fig. 4d, e). Accordingly, the narrow line of the Huygens path also shifts toward the smaller (*r*, *g*) parameter space, resulting in a totally different meta-atom selection from *p* = 340 nm to *p* = 308 nm (see Fig. 4f). In contrast, waveguide-type nanopillars are less sensitive and more robust to the surrounding environment (see Fig. S2). Nanopillar meta-atom library path selection has a much larger window (wide shaded areas in Fig. S2 vs. narrow dashed lines in Fig. 4), which is also affected when changing the refractive index from air to HSQ (the shaded area becomes narrower in Fig. S2), but not as significant as in ultrathin resonators. The same meta-atom library is still effective, while a full 2π phase modulation requires a slightly broader radius range (see Fig. S2c, f). Overall, the metasurface geometric arrangement must be carefully designed for the given membrane spacer and coating materials.

**Fig. 5:**
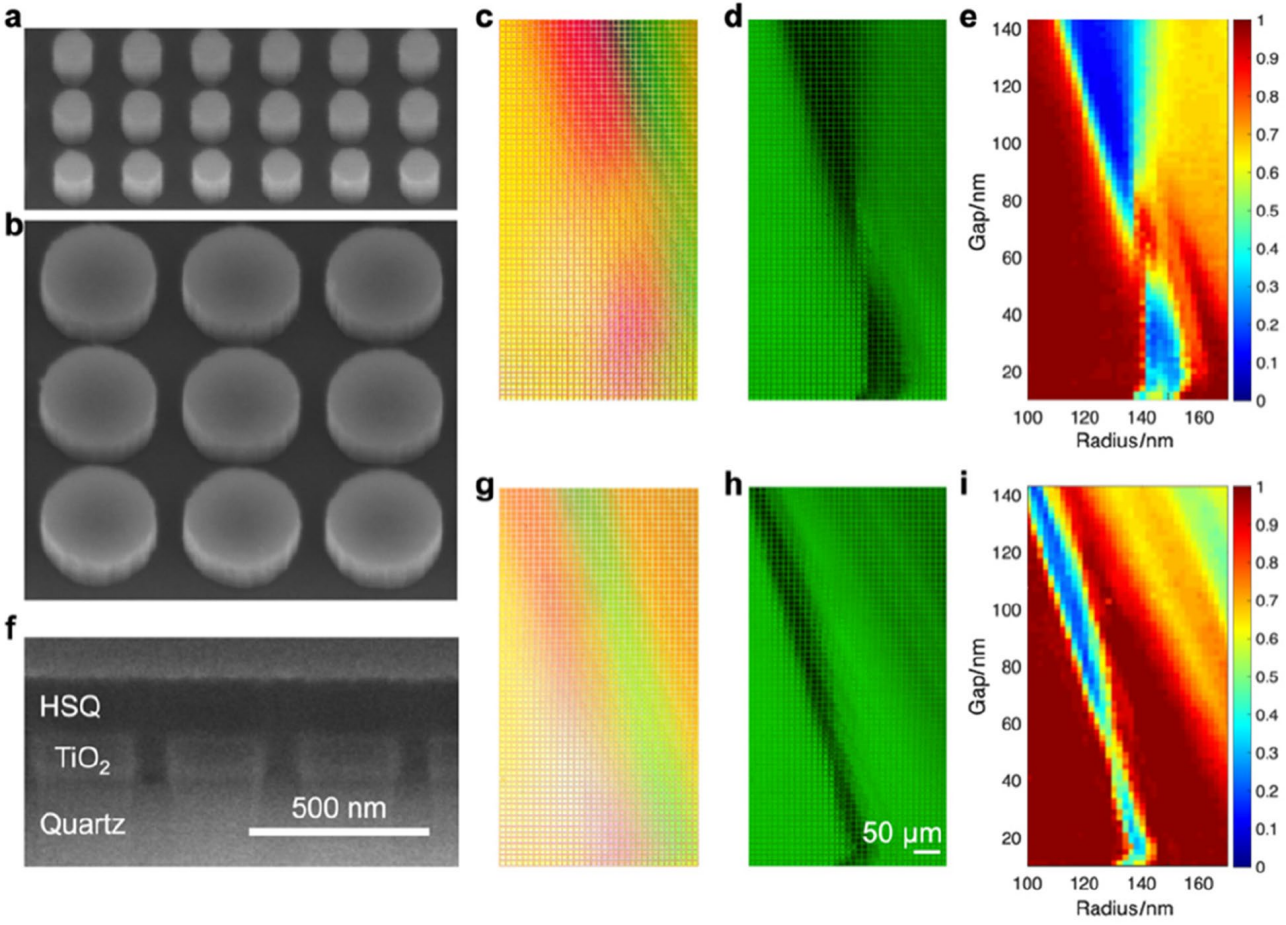
Experimental validation of the effects of the dielectric environment on ultrathin metasurfaces. Metasurface exposed to air: **a, b** SEM images of the fabricated metasurfaces with various geometric parameters. Optical image of the metasurface super-array under **c** white light illumination and **d** filtered light at the working wavelength of 532 nm. **e** Processed transmission data in the (*r*, *g*) geometric parameter space. Metasurface with the HSQ layer: **f** SEM images of the cross-section of TiO_2_ metasurfaces embedded in the HSQ layer. Optical image of the metasurface super-array under **g** white light illumination and **h** filtered light at the working wavelength of 532 nm. **i** Processed transmission data in the (*r*, *g*) geometric parameter space

Notably, our OPTIMISM process should work for both waveguide and resonator metasurfaces. Circumventing the requirement for high-aspect-ratio structures, ultrathin metasurfaces are more compatible with standard semiconductor manufacturing, which could potentially facilitate nanofabrication and transfer processes, particularly for scaling up to high-throughput levels. Therefore, we will focus on ultrathin resonator metasurfaces with more design challenges for the experimental validation (see Fig. 5). The metasurfaces were fabricated by a high-precision process based on EBL (see Fig. S3). The TiO_2_ layer was deposited on quartz substrates by atomic layer deposition. The metasurface layout was patterned in PMMA by EBL, followed by Cr deposition, lift-off, and reactive ion etching of TiO_2_, using Cr as a hard mask, which was removed by wet etching afterward. In this way, super-arrays (i.e., an array of arrays) were fabricated, where each pixel (dimensions 10 × 10 μm^2^) is a subarray of TiO_2_ nanodiscs with a constant radius and gap. Two representative SEM images of distinct subarrays are shown in Fig. 5a, b. Among different subarrays, the nanodisc radius and the gap are gradually increased along the *x* and *y* axes, respectively, following the parameter sweep performed in simulations (see Fig. 4). The super-array demonstrates resonance patterns, which are visualized as structural colors under white illumination from a Halogen lamp (see Fig. 5c), and as dark bands under filtered light at the working wavelength of 532 nm (see Fig. 5d). The experimental transmission data are extracted (see Fig. 5e), showing two dark lobes separated by the Huygens region, which match the simulated meta-atom library (see Fig. 4a) very well. Next, the same metasurface was spin-coated with a thin layer of HSQ. The HSQ-embedded metasurface is shown in the cross-sectional SEM image (see Fig. 5f). When the surrounding refractive index changes from air to HSQ, the super-array structural colors under white illumination change accordingly (see Fig. 5g). The dark bands under filtered green light become narrower and shift toward smaller (*r*, *g*) pairs in the geometric parameter space (see Fig. 5h). The processed transmission data (see Fig. 5i) are also in good agreement with simulations (see Fig. 4d).

### Metasurface inverse design considering the dielectric environment

**Fig. 6:**
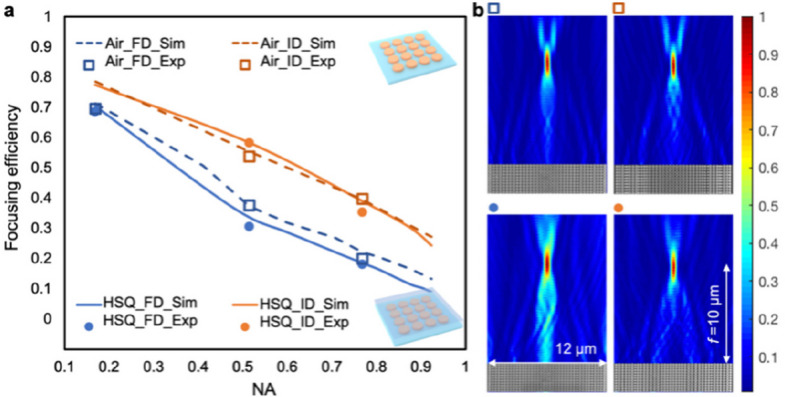
Role of the dielectric environment in improving metasurface performance by inverse design versus forward design. **a** Comparison of focusing efficiency versus NA of cylindrical lenses by both forward and inverse designs, in dielectric environments of air and HSQ layer. **b** SEMs of metalenses with NA = 0.51 (lens diameter 12 μm, focal length 10 μm) and the corresponding measured light intensity distributions along the axial direction (*x*–*z* planes) for both forward and inverse designs, in dielectric environments of air and HSQ layer. HSQ results are adopted from our previous work [29]. Exp, experiment; FD, forward design; ID, inverse design; Sim, simulation

For ultrathin resonant metasurfaces both exposed to air and embedded in HSQ, the simulated meta-atom libraries were validated experimentally and can be used for metasurface design through the conventional forward approach, i.e., reproducing optical phase profiles with library search from a predetermined database. Importantly, it was found in our previous work [29] that the forward design is only applicable to independent waveguide-type metasurfaces. For strongly coupled resonant metasurfaces where the local arrangement deviates from the predetermined homogeneous configurations, the forward design leads to performance degradation, which can be improved by inverse design strategies (e.g., based on an evolutionary algorithm). Here, we further investigate the role of dielectric environments in improving metasurface performance by inverse design. We designed and simulated TiO_2_ cylindrical metalenses (*t* = 115 nm) for green light (*λ* = 532 nm) with various numerical apertures (NAs) through both forward and inverse designs (see Fig. 6). Among all the NAs, we fabricated the designs for NAs of 0.17, 0.51, and 0.77 (NA = 0.17 corresponding to a lens diameter of 12 μm and a focal length *f* of 35 μm, and the rest corresponding to a lens diameter of 12 μm and 24 μm, respectively, with a constant *f* of 10 μm), which demonstrated diffraction-limited focusing (see Fig. S4). In Fig. 6a, the focusing efficiencies of metalenses exposed to air (dashed lines represent simulations and boxes represent data points of experiments) are compared with those embedded in HSQ layers, which are adopted from our previous work (solid lines represent simulations and circles represent data points of experiments) [29]. Overall, the experimental results are in good agreement with the simulations. The forward-designed metalenses exposed to air exhibit slightly higher focusing efficiencies than those embedded in HSQ layers, probably because they provide a larger refractive index contrast between the meta-atom and environment, along with lower non-local interactions. Nevertheless, after inverse design, the optimized metalenses embedded in HSQ layers can be improved to achieve focusing efficiencies comparable to, or even exceeding, those of metalenses exposed to air, demonstrating the advantage of inverse design in leveraging non-local interactions.

Taking NA = 0.51 as an example, Fig. 6b shows the SEMs of fabricated metalenses and measured light intensity distributions along the axial direction (*x*–*z* planes) for both forward and inverse designs, in dielectric environments of both air and HSQ, respectively. The nanodiscs are in a periodic (constant pitch) arrangement in the forward designs (SEMs in Fig. 6b, left). Light intensity distributions show diverging directions and satellite peaks, reducing the light reaching the focal point, where the metalens embedded in HSQ (see Fig. 6b, bottom left) is even worse than that exposed to air (see Fig. 6b, top left). In contrast, inverse designs are in aperiodic arrangements with more empty spaces (SEMs in Fig. 6b, right). Most of the light is directed toward the focal point, providing a higher focusing efficiency, where the metalens embedded in HSQ (see Fig. 6b, bottom right) is similar to that exposed to air (see Fig. 6b, top right).

## Conclusions

In conclusion, rather than metasurfaces fabricated on bulky substrates, the MISM platform enables metasurface transfer and integration with non-conventional and nonplanar substrates, as well as electronic/photonic devices. We summarize multiple approaches to create MISM platforms with a variety of membrane and sacrificial layer materials and configurations, which are either suspended on-chip or fully released at the liquid–air interface. The suspended MISMs can be transferred via a contact printing process using elastomer stamps or assisted by adhesives. In this work, we demonstrate the OPTIMISM technology, demonstrated by a direct punching process without using any intermediate carriers or adhesives, which is particularly suitable for integration on optical fiber tips. The versatility of OPTIMISM will help overcome current metasurface transfer limitations in material and geometry (nanohole metasurfaces, plasmonic metasurfaces, thick elastomer membranes). Moreover, environmentally friendly sacrificial layers and etching solutions were selected over aggressive chemicals. Metasurface transfer and integration were performed on a single optical fiber tip. Nevertheless, in the literature, “free-floating” MISMs have been fabricated at the wafer scale [4], while scalable integration has been demonstrated with multiple fiber arrays in parallel using adhesives [27]. The MISM array (see Fig. 3) indicates that our OPTIMISM technology can also be scaled up to high-throughput levels. In the future work, we will quantitatively investigate the mechanical stability, optical performance, transfer yield, alignment accuracy, and batch-to-batch reproducibility. Meta-fibers will be packaged with a protective sheath and characterized in biological media for imaging and sensing applications.

Without high-aspect-ratio structures, ultrathin resonant metasurfaces could potentially facilitate scalable nanofabrication and transfer processes. Therefore, we investigated the influence of membrane and coating materials on ultrathin metasurface design based on both library search and inverse design strategy. Through both simulations and experiments, we found that the optical resonance, transmission and phase outputs of meta-atom libraries are very sensitive to the dielectric environment, which should be carefully considered during the design process. Compared to the dielectric environment of air, membrane and coating materials reduce the refractive index contrast between the meta-atom and environment, thereby increasing non-local interactions and leading to performance degradation in conventional forward design. Nevertheless, inverse design strategies leverage non-local interactions to compensate for degradation and improve the performance, which can be further enhanced by developing novel machine learning and topology optimization algorithms. Contrary to medium-switchable metasurfaces [35], inverse design strategies could also potentially reduce the sensitivity of metasurfaces to the dielectric environment to a limited extent. Therefore, it is more practical and straightforward to simply fix the dielectric environment by using the membrane and coating materials for metasurface design.

Finally, the MISM platform enables metasurface transfer and integration that is universal and versatile with various receivers (e.g., optical fibers, electronic/photonic devices, dynamic actuators), membrane materials (e.g., Si, Al_2_O_3_, PMMA, SU-8, HSQ), and configurations, for both resonator and waveguide-type metasurfaces. The feasibility of high-aspect-ratio metasurfaces has been demonstrated by their integration with elastomers [11, 12]. Taking advantage of the outstanding mechanical properties of dielectric membranes (e.g., Al_2_O_3_) [36], the MISM platform offers enhanced potential for mechanical actuation and hybrid integration with microelectromechanical systems (MEMS) and nanoelectromechanical systems (NEMS), beyond what has recently been demonstrated using FIB [37].

## Methods

### Metasurface membrane transfer and integration

Multiple metasurface fabrication and transfer processes were developed in this work. Dielectric (TiO_2_) metasurfaces were fabricated on a GeO_2_ sacrificial layer and then embedded in a PMMA layer. The MISM was released in water following the processes described in our previous work [28]. Alternatively, plasmonic or polymer metasurfaces were fabricated on an SU-8 thin film on a sacrificial layer of OmniCoat. The OmniCoat was spin-coated at 3 000 rpm and then baked at 200 °C for 1 min. SU-8 2010 was spin-coated at 8 000 rpm to reach a 7-µm thickness, soft-baked at 65 °C for 2 min followed by 95 °C for 2 min, and exposed to pattern the aperture and tether structures using a μMLA Maskless Aligner (Heidelberg Instruments) at 600 mJ/cm^2^. Post-exposure baking was performed at 65 °C for 1 min followed by 95 °C for 1 min. The sample was developed in an SU-8 developer for 1 min, rinsed in isopropyl alcohol (IPA), dried with N_2_, and hard-baked at 150 °C for 3 min. The MISM sample was then released in Microposit MF-319 overnight. The floated sample was transferred to a dish with DI water using a folded aluminum foil boat. A 3-mm-diameter hole was drilled in a glass slide using a glass drill under flowing water. The glass slide was then placed in a dish, and the MISM sample was carefully aligned to the center of the hole. Water was removed with a pipette until the sample lay flat on the slide, which was dried completely on a 40 °C hotplate. The MISM was suspended over the hole, ready for optical fiber punching. The optical fiber was attached to a translation stage and aligned to the MISM aperture under a Leica M60 stereo microscope. The fiber was perpendicular to the aperture and aligned to its center. The stage was carefully moved upward until the fiber tip fully punched through the SU-8 layer. The fiber integrated with the metasurface was then imaged by SEM (Zeiss GeminiSEM 300).

### Metasurface optical simulations

The optical simulations were carried out and cross-validated using commercial software (COMSOL, Lumerical). A single TiO_2_ meta-atom was modeled with periodic boundary conditions in both *x* and *y* directions. The geometric parameters of disc radius and gap between discs were swept to build a meta-atom library database. The incident light was along the *z*-axis, with polarization along the *y*-axis. Both transmission and phase outputs were collected for nanodiscs exposed to air or embedded in an HSQ layer, respectively (see Fig. 4). The transmission was defined as the transmitted power normalized by the source power. For device simulation, a whole metalens was modeled to record the *x*–*z* intensity distribution of the lens (see Fig. 6b), which also provided focal spot line profiles for the evaluation of focusing efficiency (see Fig. 6a). The inverse design strategy using the evolutionary algorithm followed the methods reported in our previous work [29].

### Optical characterization

The metasurfaces were imaged under an inverted microscope (Olympus IX73) by white light (with and without a 532-nm filter) from a Halogen lamp (see Fig. 5). A green laser (wavelength 532 nm, Opto Engine) was introduced from the top of the inverted microscope via a home-built set-up for metalens characterization. Image stacks were obtained while moving the stage in the *z*-direction automatically (Prior ES10ZE Focus Controller) and then processed to obtain the *x*–*z* intensity distribution (see Fig. 6b) and focal spot line profiles, which were used for the evaluation of focusing efficiency.

## Supplementary Information

Supplementary Material 1.

## Data Availability

All data generated or analyzed during this study are included in this published article and its supplementary information files.
