# Supplementary material for: Transfer, integration, and inverse design of metasurfaces in suspended membranes
Source: Moore More. 2026 Feb 16;3(1):1. doi: 10.1007/s44275-026-00041-y (PMC12939920; doi:10.1007/s44275-026-00041-y)
Supplement: Supplementary file 1 — Supplementary Material 1. [file 44275_2026_41_MOESM1_ESM.docx]

**Transfer, Integration, and Inverse Design of Metasurfaces in Suspended Membranes**

Nanzhong Deng^1,2^ · Yue Xiao^1,2^ · Srilok Srinivasan^3^ · Subramanian K. R. S. Sankaranarayanan^3,4^ · Xu Zhang^5^ · David Czaplewski^3^ · Daniel Lopez^6^ · Haogang Cai^1,2,7,^*

^1^ Tech4Health Institute, New York University Grossman School of Medicine, Queens, NY 11101, USA.

^2^ Department of Biomedical Engineering, New York University, Brooklyn, NY 11201, USA.

^3^ Center for Nanoscale Materials, Argonne National Laboratory, Lemont, IL, 60439, USA.

^4^ Department of Mechanical and Industrial Engineering, University of Illinois, Chicago, IL 60607, USA

^5^ Department of Electrical and Computer Engineering, Carnegie Mellon University, Pittsburgh, PA 15213, USA

^6^ Department of Electrical Engineering & Materials Research Institute, The Pennsylvania State University, University Park, PA, 16802, USA

^7^ Department of Radiology, New York University Grossman School of Medicine, New York, NY, 10016 USA

*corresponding author: haogang.cai@nyu.edu


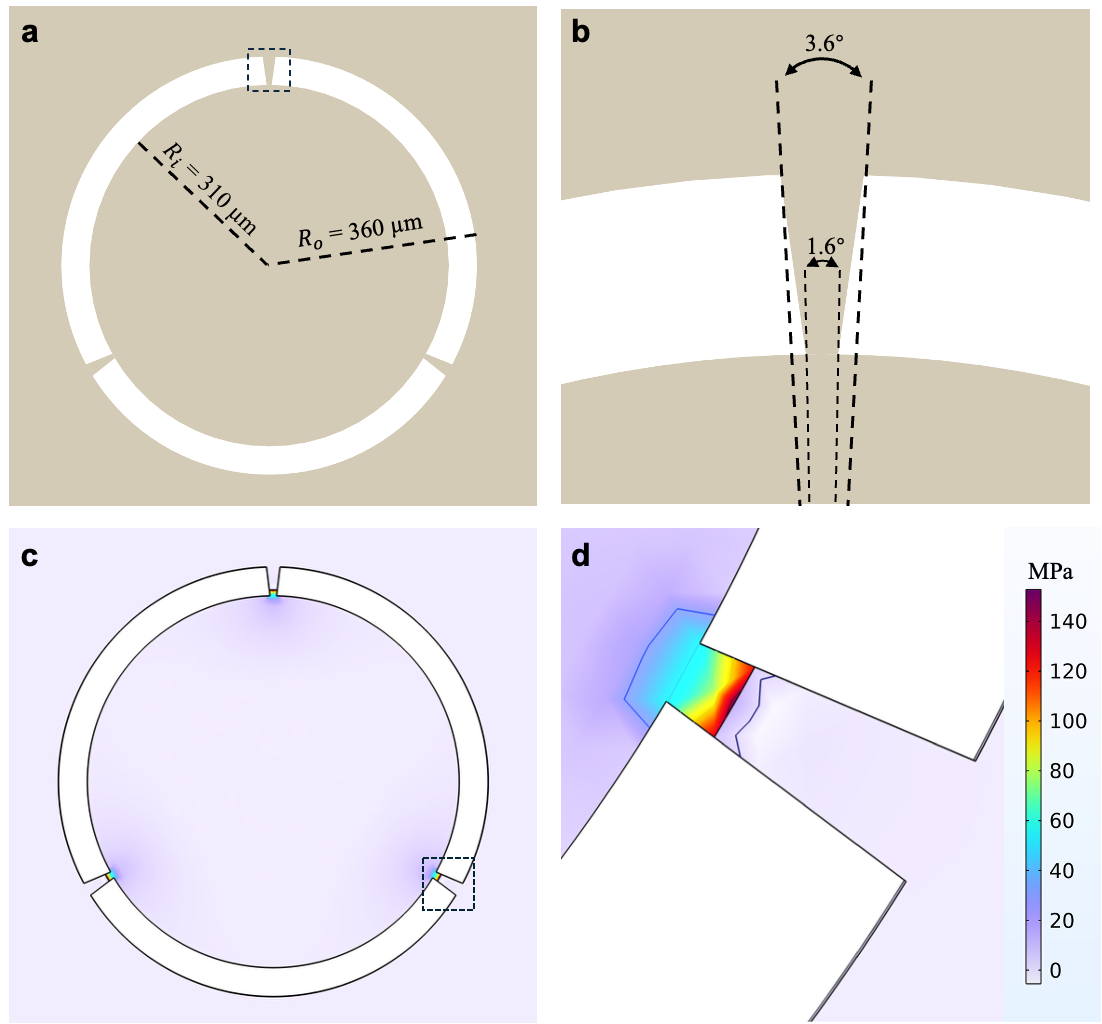


**Figure. S1 Aperture and tether structure layouts and mechanical simulations.** (a) Aperture and tether structure layouts with geometric parameters (b) Zoom-in of the tether structure layouts. (c) COMSOL simulation of pressure distribution on a SU-8 membrane when an optical fiber is punched through. (d) Zoom-in of the pressure distribution in the tether structure. The pressure is mainly concentrated on the thin tether connection parts touching the fiber edge, which easily break during the punching process without affecting the metasurfaces on the membrane.

**Table. S1 Geometric parameter and mechanical propertiess of the SU-8 membrane**

| Thickness | 10 μm |
| --- | --- |
| Density | 1.20 x 10^3^ kg·m^-3^ |
| Young’s modulus | 4.02 GPa |
| Poisson’s ratio | 0.22 |
| Bulk modulus | 2.39 GPa |
| Shear modulus | 1.65 GPa |
| Tensile Strength | 90 MPa |
| Force | 5 mN |


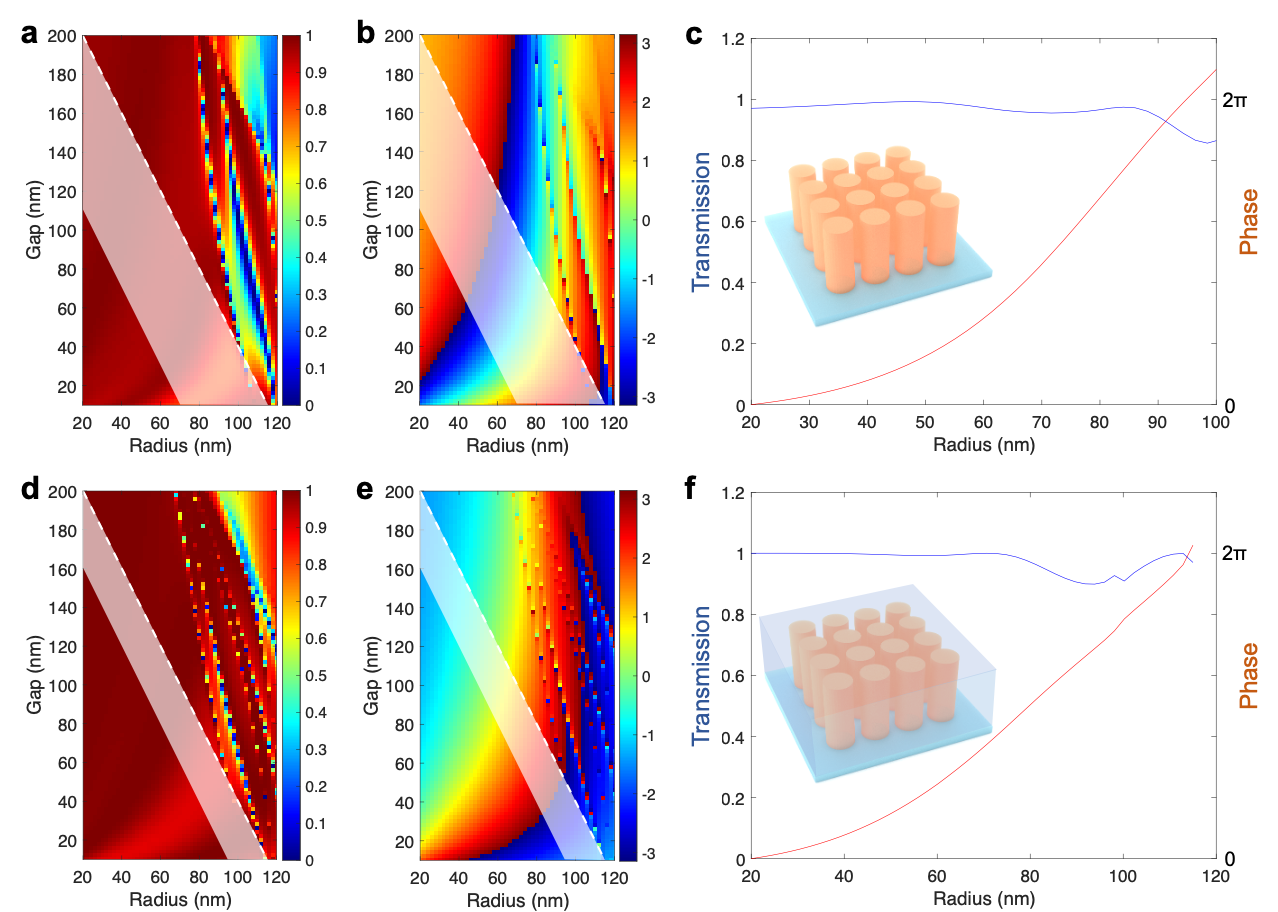


**g**

Figure S**2**. **Dielectric environment effects on high-aspect-ratio waveguide-type metasurfaces.** Simulation results of the transmission and phase outputs of a uniform wavefront of light after propagation through a homogeneous and periodic square array (circular TiO_2_ nanopillars, thickness t = 600 nm, on glass substrate). Metasurface exposed in air without any coating: (a) transmission and (b) phase as a function of the pillar radius and edge-to-edge gap; (c) transmission and phase for a meta-atom library with constant pitch of 240 nm and varying pillar radius (following the path marked by white dashed lines in a, b). Metasurface with HSQ coating: (d) transmission and (e) phase as a function of the pillar radius and edge-to-edge gap; (f) transmission and phase for the same library with constant pitch (240 nm) and varying pillar radius. (g) TiO_2_ refractive index data measured by ellipsometry and used in the simulation.


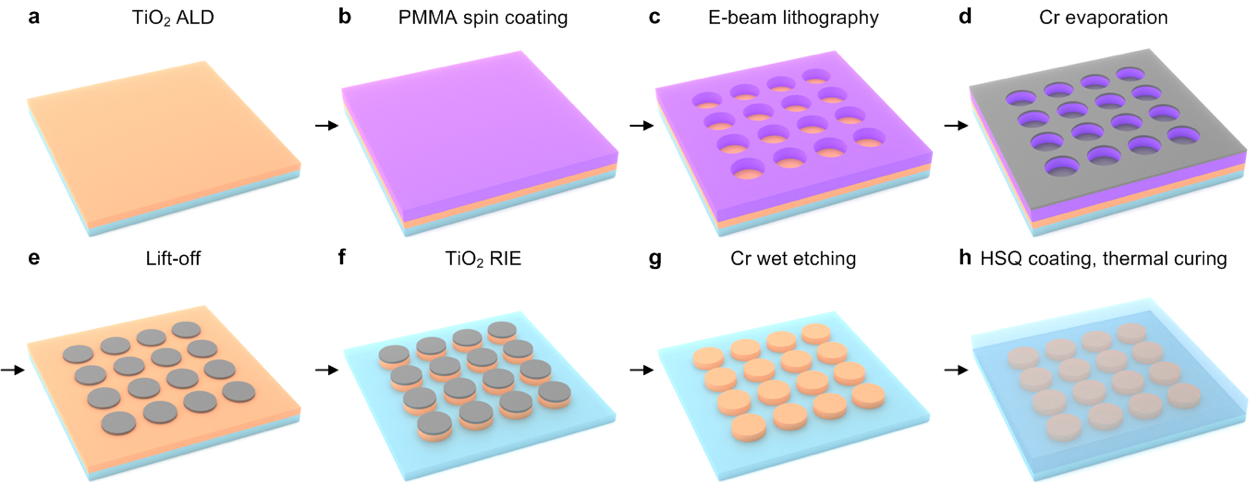


**Figure S3. Schematic diagram of the nanofabrication process.** (a) TiO_2_ layer is deposited on quartz substrate by atomic layer deposition (ALD: precursor TDMATi, at 150 °C), and processed by rapid thermal annealing (RTA: at 475°C for 30 min in O_2_) in order to increase its refraction index. (b) The sample is spin-coated with a bilayer of PMMA (MW 495K and 950K, Microchem). (c) The sample is patterned by a single step of e-beam lithography (JEOL JBX-9300FS). After exposure, the Au conductive layer is removed by wet etching, then the sample is developed in MIBK/IPA 1:3 at 4 °C with ultrasonication for 1 min. (d) A 10 nm Cr layer is deposited by e-beam evaporation, (e) leaving Cr discs after lift-off in Remover 1165 at 75 °C. (f) The pattern is transferred into the underlying TiO_2_ layer by reactive ion etching (RIE: SF_6_, 12 sccm, 6 mTorr, 150 W). (g) The Cr masks are removed using an aqueous etchant, leaving TiO_2_ nanodisc arrays on the quartz substrate. (h) The sample is spin-coated with HSQ and thermally cured (RTA: at 400°C for 15 min in N_2_).


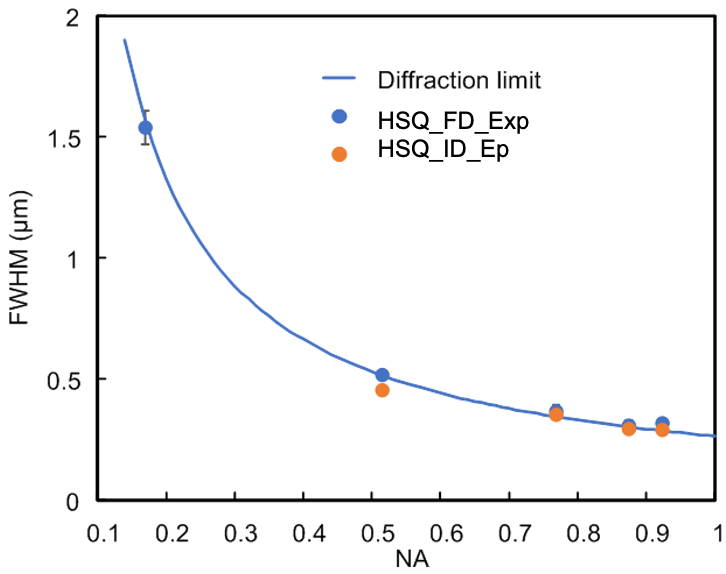


**Figure S4. Experimental results of diffraction-limited focusing.** Focal spot size (FWHM) vs. numerical aperture (NA). For both forward (FD) and inverse designs (ID), the measured focal spots are diffraction-limited for all NAs.

**Inverse design evolutionary algorithm, computational cost and limitations**

The inverse design strategy based on an evolutionary algorithm is available in GitHub

(<https://github.com/Srilok/GA-for-inverse-design>). All the cases were converged within 250 generations, although an improved design close to (within 3% or less than) the converged best is obtained much earlier than that, typically around 60-80 generations. The computational cost will scale with the simulation area. Therefore, the major challenges in the scalability of our inverse design strategy are not only to enlarge the metasurface area (currently it is only a diameter of 10 μm), but also to extend the configuration from 1D cylindrical lenses to 2D spherical lenses.
